# Periprosthetic tibial fractures in total knee arthroplasty – an outcome analysis of a challenging and underreported surgical issue

**DOI:** 10.1186/s12891-018-2250-0

**Published:** 2018-09-11

**Authors:** Anna Janine Schreiner, Florian Schmidutz, Atesch Ateschrang, Christoph Ihle, Ulrich Stöckle, Björn Gunnar Ochs, Christoph Gonser

**Affiliations:** 1 BG Trauma Center Tübingen, Eberhard Karls University Tübingen, Schnarrenbergstrasse 95, 72076 Tübingen, Germany; 2 Department of Orthopaedic Surgery, Physical Medicine and Rehabilitation, University of Munich (LMU), Marchioninistraße 15, 81377 Munich, Germany; 3 Department of Orthopedics and Trauma Surgery, Medical Center, Faculty of Medicine, Albert-Ludwigs-University of Freiburg, Hugstetter Str. 55, 79106 Freiburg, Germany

**Keywords:** Periprosthetic fractures, Total knee arthroplasty, Felix, Malalignment

## Abstract

**Background:**

Periprosthetic fractures after total knee arthroplasty (TKA) are an increasing problem and challenging to treat. The tibial side is commonly less affected than the femoral side wherefore few studies and case reports are available. The aim of this study was to analyze the outcome of periprosthetic tibial fractures and compare our data with current literature.

**Methods:**

All periprosthetic tibial TKA fractures that were treated at our Level 1 Trauma Center between 2011 and 2015 were included and analyzed consecutively. The Felix classification was used to assess the fracture type and evaluation included the radiological and clinical outcome (Knee Society Score/KSS, Oxford Knee Score/OKS).

**Results:**

From a total of 50 periprosthetic TKA fractures, 9 cases (7 female, 2 male; 2 cruciate retaining, 7 constrained TKAs) involving the tibial side were identified. The mean age in this group was 77 (65–85) years with a follow-up rate of 67% after a mean of 22 (0–36) months. The Felix classification showed type IB (*n* = 1), type IIB (*n* = 2), type IIIA (*n* = 4) and type IIIB (*n* = 2) and surgical intervention included ORIF (*n* = 6), revision arthroplasty (*n* = 1), arthrodesis (*n* = 1) and amputation (*n* = 1). The rate of adverse events and revision was 55.6% including impaired wound healing, infection and re-fracture respectively peri-implant fracture. Main revision surgery included soft tissue surgery, arthrodesis, amputation and re-osteosynthesis. The clinical outcome showed a mean OKS of 29 (19–39) points and a functional/knee KSS of 53 (40–70)/41 (17–72) points. Radiological analyses showed 4 cases of malalignment after reduction and plate fixation.

**Conclusions:**

Periprosthetic tibial fractures predominantly affect elderly patients with a reduced bone quality and reveal a high complication rate. Careful operative planning with individual solutions respecting the individual patient condition is crucial. If ORIF with a plate is considered, restoration of the correct alignment and careful soft tissue management including minimal invasive procedures seem important factors for the postoperative outcome.

## Background

Periprosthetic fractures constitute an upcoming challenge in revision arthroplasty. Reasons include increasing numbers of total knee arthroplasty (TKA), longer life expectancy and implant survival as well as patient related risk factors such as osteoporosis and sarcopenia [1].

The overall incidence of periprosthetic fractures after TKA is estimated to range between 0.3 to 2.5% [2–4]. The vast majority involves the distal femur (2%) whereas the proximal tibia is less frequently affected (0.3–0.5%) and thus has received little attention in the past [5]. The intraoperatively rate of periprosthetic TKA fractures is given with about 4% [6], but is likely to be underreported. Nevertheless, the majority of periprosthetic TKA fractures usually occur 2–4 years postoperatively after TKA and increases after revision TKA up to 38% [4].

Risk factors for periprosthetic TKA fractures include patient related factors like osteoporosis, age, female sex, revision and osteolysis. Specific surgical risk factors are the use of long tibial stems, cementless press-fit fixation, malalignment or malrotation of the tibial component, previous osteotomy e.g. of the tibial tuberosity and osseous defects in revision arthroplasty [5, 7, 8]. Besides, periprosthetic tibial fractures seem to be closely related to the implant design.

Periprosthetic fractures are a life-threatening condition for many of the predominantly elderly patients with a previous study reporting a one-year mortality rate between 11 to 44.8% [9, 10]. Therefore, the main object is to achieve early mobilization with a good functionality to reduce mortality [11]. Further aims include restoration of leg axis, bone-implant union and a stable joint which is influenced by patients’ general condition as well as the type of TKA.

In contrast to periprosthetic TKA fractures of the femur, only few studies with limited numbers of patients have analysed periprosthetic fractures of the tibia. Therefore, the aim of this study was to analyse functional and radiological outcomes after periprosthetic TKA fractures and compare our data with those reported in the current literature providing present information to better anticipate prospective developments in revision arthroplasty in especially geriatric patients in the long-term.

## Methods

This consecutive analysis included all patients who were initially treated or revised for a tibial periprosthetic TKA fracture in the Department of Arthroplasty at our Level 1 Trauma Centre between 2011 and 2015. Classification of the periprosthetic tibial fractures was done according to the widespread Felix classification which is also known as the Mayo classification [12]. The classification was introduced by Felix et al based on an analysis of 102 periprosthetic tibial fractures [13] and includes 4 types related to the major anatomic pattern (I = tibial plateau, II = adjacent to the stem, III = distal to the prosthesis, IV = tibial tubercle) as well as 3 subcategories regarding fixation and time of fracture (A = prosthesis well fixed, B = loose prosthesis, C = intraoperative) [13]. While a loose prosthesis (Felix type B) usually indicates revision arthroplasty, Felix A and C fractures may be addressed by operative or non-operative fracture management [12].

All fractures were analysed radiologically and if the patients were available also examined clinically within the study. The standardized clinical examination was performed by one examiner comprising range of motion, pain, stability of the affected knee and palpation of the TKA site as well as outcome rating with the established Knee Society Score [14] and Oxford Knee Score [15, 16].

Radiological examinations consisted of standardized radiographs of the knee and/or the lower leg in two planes (anteroposterior and lateral views) and were analysed for tibial malalignment, union rate and implant failure. A deviation on fracture reduction and/or fixation of ≥5° in both planes as well as a deviation ≥5° of the 90° tibial slope angle were rated pathological. Malalignment regarding the tibial slope was expressed as exceeding tilt angle of the tibial plateau. Measurements were performed with the commercially available evaluation tool mediCAD® (HECTEC GmbH, Landshut, Germany) which is imbedded in the clinical image data base IMPAX® (Agfa HealthCare GmbH, Bonn, Germany).

Apart from radiological and outcome measurements, additional patient information such as previous history and surgery or any other adverse events before and after surgery were captured from the digital patient charts of our hospital. The last preceding medical procedure before the periprosthetic fracture was rated as index surgery which could either be primary or revision arthroplasty.

In case of loss of follow-up because of decease of the patient or drop-out the latest patient file data and radiographs available were analysed retrospectively. Insufficient data was graded as exclusion criteria. Patients suffering from dementia, with insufficient knowledge of the study language or incapability of participation in the study due to severe health conditions e.g. were excluded as well. The study protocol was approved by the local ethics committee (622/2015B02) and patients gave their written consent to participate in the study. Statistical analysis including patient demographics and data displayed as mean, standard deviation and range was performed with Microsoft Excel 2016 (Microsoft Corporation, Redmond, USA).

## Results

**Fig. 1:**
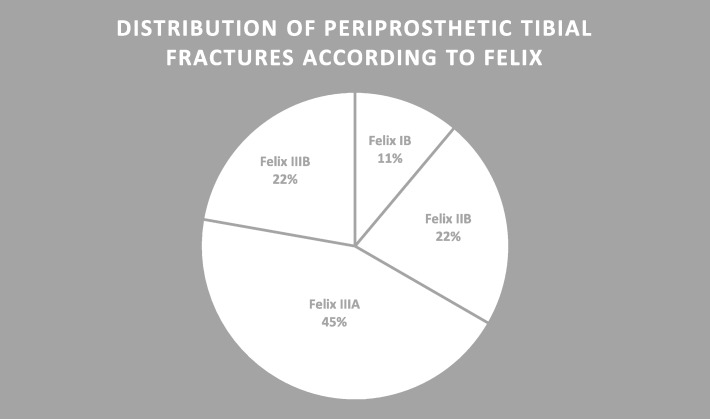
Distribution of periprosthetic fractures according to Felix (Felix IB *n* = 1, IIB *n* = 2, IIIA *n* = 4, IIIB *n* = 2)

Overall, 50 periprosthetic fractures associated to TKA were identified, from which 9 cases (18%) showed a periprosthetic tibial TKA fracture. Figure 1 displays the distribution according to the Felix classification. No case had to be excluded according to our exclusion criteria. The cohort (7 female, 2 male) showed a mean age of 77.1 ± 6.0 (65–85) years and a BMI of 28.5 ± 3.7 (25–36) kg/m^2^ at the time of surgery due to their periprosthetic fracture. The total mean follow-up was 22.3 ± 10.5 (0–36) months with a follow-up rate of 67% (2 drop-outs and 1 death due to internal medical reasons). All patients suffered from comorbidities with a mean of 2.3 ± 1.6 (0–5) orthopaedic and 4.6 ± 2.5 (1–10) internal side diagnoses. Mean time between index surgery and periprosthetic tibial fracture was 59.7 ± 64.1 (0.25–209) months. Retrospectively, there were 2 intraoperative fractures which were diagnosed with a delay of 6 days and 4 months, respectively. Time period between occurrence of the periprosthetic tibial fracture and revision surgery was 1.1 ± 1.6 (0–5) months. The primary TKA was performed between 1997 and 2015.

Surgical history of the patients was uneventful in 3 cases with 6 cases having a low grade periprosthetic joint infection (PJI), aseptic loosening, mechanical failure, wound healing disorder, unicompartimental knee arthroplasty (UKA) and patellectomy with partly several revisions before index surgery. In summary, 67% of all patients had 1.3 times preceding surgery. The cohort comprised 2 cruciate retaining prostheses and 7 semi- or fully constrained total knee arthroplasties.

**Fig. 2:**
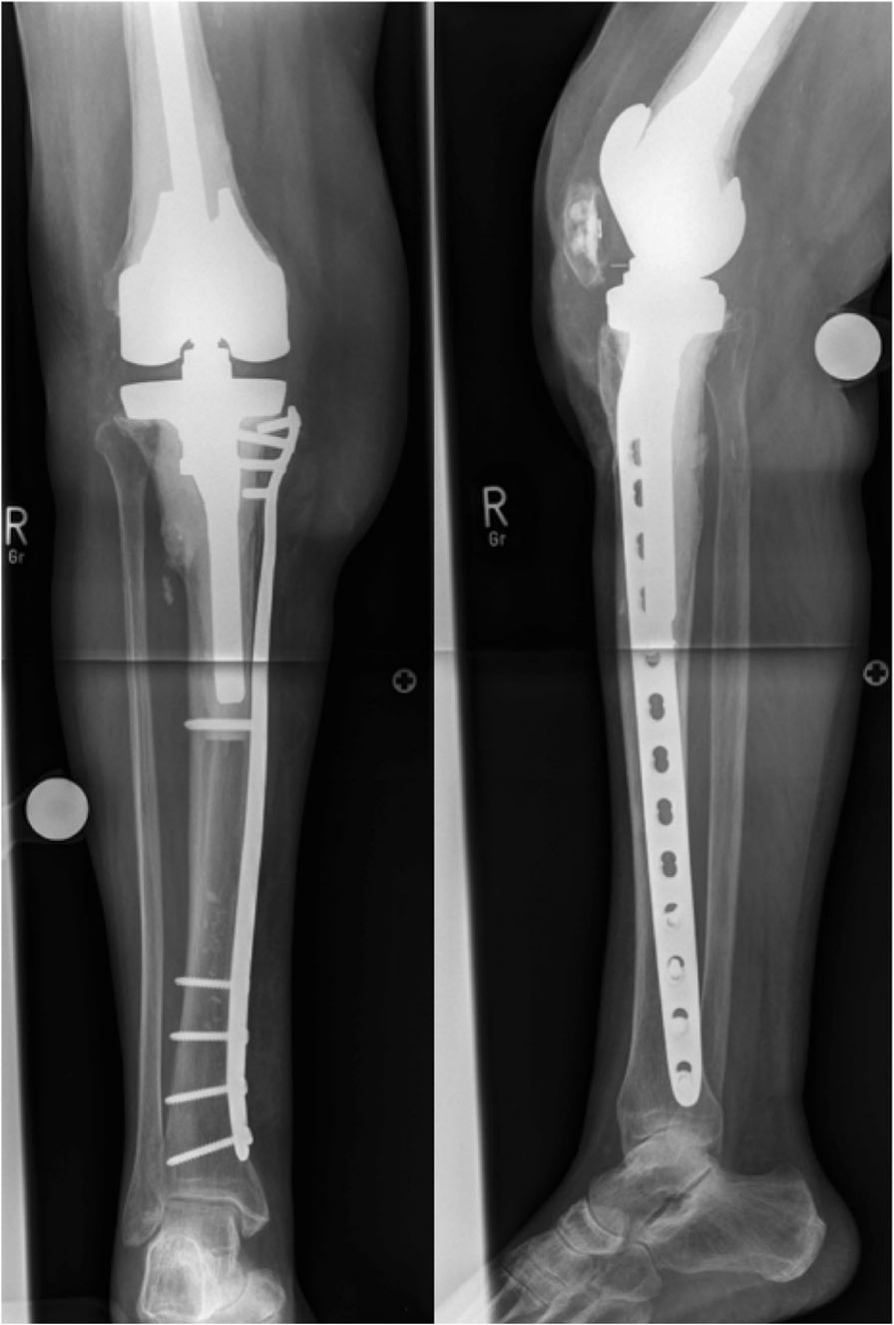
ORIF with a long medial plate in a periprosthetic tibial fracture around a hinged TKA

There were 7 cases of low energy trauma and 2 cases related to osteolysis causing the periprosthetic tibial fracture. Mean duration of hospital stay was 22.8 ± 13.0 (8–51) days and mean duration of surgery was 109.9 ± 41.4 (63–169) minutes with a transfusion rate of 43%. Surgery due to the periprosthetic tibial fracture comprised *n* = 6 open reduction and internal fixation (ORIF), *n* = 1 revision arthroplasty, *n* = 1 arthrodesis and *n* = 1 amputation (Fig. 2).

One case (Felix type IIIB) had been treated with ORIF at another hospital first and was revised at our hospital due to PJI with loosening of the TKA. The type IB fracture was successfully revised with revision arthroplasty. This patient also survived an intraoperative lung arteries embolism. One of the type IIB fractures resulted in an amputation due to an extensive PJI with a wound healing disorder in the course. The other type IIB fracture was treated with ORIF and finally lead to arthrodesis due to wound healing disorders, loosening and infection. One of the IIIA fractures was successfully treated with ORIF with a wound healing disorder that could be handled conservatively. The other type IIIA fracture was followed by 5 revisions including re-ORIF with additional autologous bone grafting following re-fracture, non-union, wound healing disorders and a peri-implant fracture. The other 2 type IIIA fractures showed no adverse events after osteosynthesis. One of the type IIIB fractures was treated with ORIF followed by 6 revision surgeries due to implant failure, infection and wound healing disorder and finally resulting in amputation. The other type IIIB fracture was successfully treated with arthrodesis. In total, the rate of adverse events as well as revision was 55.6% (*n* = 5 each). Osteosynthesis was applied by the majority (66.7%, *n* = 6). Amputation as well as arthrodesis were treatment options in the first place due to an infectious constellation in one case and extensive bone loss in the other case always also regarding patients’ age and demands.

Main complications were wound healing disorders (41.7%), infection (16.7%) and re-fracture or peri-implant fracture (16.7%) with 0.5 adverse events per patient. Loosening, implant failure and non-union occurred in 8.3% each. Main revision surgery included soft tissue surgery (28.6%), arthrodesis (28.6%), amputation (14.2%) and re-osteosynthesis (28.6%) with 1.5 revisions per patient. Table 1 gives an overview of the treatment of the periprosthetic fractures of the tibia in our cohort.

**Table 1:** Overview of periprosthetic tibial fractures regarding treatment, adverse events and revision surgery

Felix	n	Treatment	Adverse Events	Revision Surgery
IB	1	Revision Arthroplasty	–	–
IIB	2	ORIF	Wound healing disorder, infection, loosening	Arthrodesis
		Amputation	Wound healing disorder	Soft tissue revision
IIIA	4	ORIF	Wound healing disorder	–
		ORIF	Re-fracture, non-union, wound healing disorder, peri-implant fracture	Several Re-ORIF, autologous bone grafting
		ORIF	–	–
		ORIF	–	–
IIIB	2	ORIF	Implant failure, infection, wound healing disorder	Several Re-ORIF, amputation with soft tissue revision
		Arthrodesis	–	–

Mean Oxford Knee Score was 28.8 ± 6.6 (19–39) points. The functional Knee Society Score and the knee Knee Society Score showed a mean of 53.3 ± 13.7 (40–70) points and 41.3 ± 17 (17–72) points, respectively. Clinical examination comprising pain, stability of the affected knee and palpation of the site showed no relevant results. Mean range of motion at the time of follow-up was 0–0-100°.

**Fig. 3:**
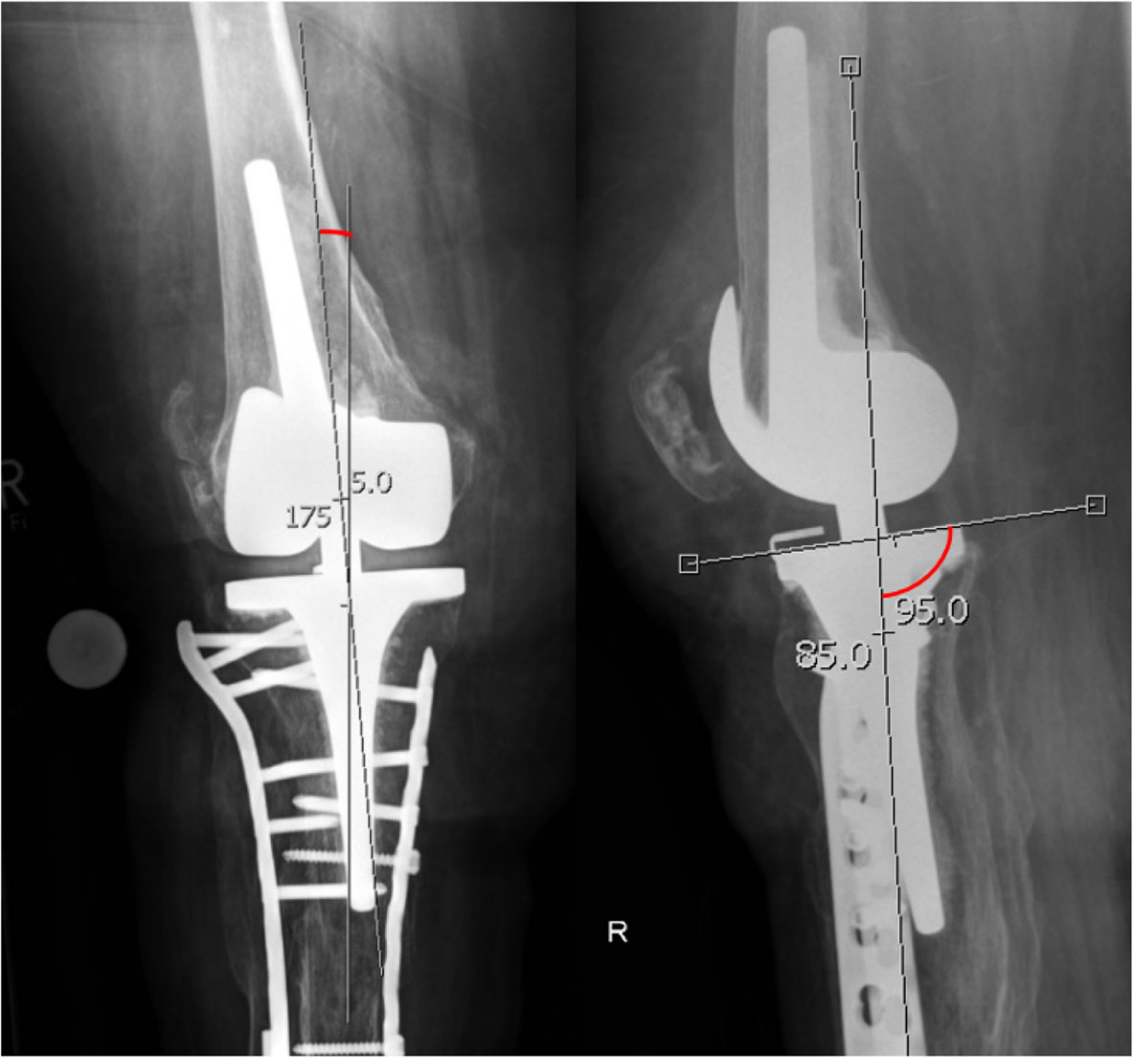
Malalignment after double plate fixation of a periprosthetic tibial fracture (Frontal plane: 5° varus malalignment; sagittal plane: 5° anterior tibial slope)

Radiological evaluation revealed 2 cases of malalignment after ORIF with a plate in the coronal plane (6° and 7° varus malalignment) as well as 2 cases of malalignment in both planes (both 5° malalignment in the frontal plane as well as 8° and 5° in the sagittal plane). Figure 3 shows a case with combined malalignment in both planes. No case showed an isolated malaligned tibial slope in comparison to isolated malalignments in the frontal plane after plate fixation as described above. The other cases were *n* = 4 amputations/arthrodesis and *n* = 1 correct alignment. There was no implant failure and the healing rate was 100% (for *n* = 5 ORIF) at the time of follow-up.

## Discussion

Periprosthetic tibial fractures represent a rare but potentially fatal complication after TKA. In this study, we present a consecutive series of tibial TKA fractures, confirming the challenging treatment associated with a high complication rate. To the best of our knowledge, this is the only current study on PubMed evaluating the treatment and outcome of tibial Felix type fractures after TKA. Only Kim et al. reported a series with minimally invasive plate osteosynthesis (MIPO) [17]. Like previous reports, our data are limited by the small case number which does not allow present statistical statements so far.

In the original report of Felix et al., type I fractures represent the main fracture type (*n* = 61/102), while Felix type III fractures (n = 6/9) were the most frequent ones observed in ours [13]. Furthermore, we recorded no type 4 fracture which complies with the low incidence (*n* = 2/102) as reported before [13]. Accordingly, most tibial TKA fractures occurred in our cohort postoperatively (*n* = 7/9). Felix et al. further described a predominate pattern of type IB and IIB fractures that were usually treated by revision surgery [13]. In our series, these Felix types (IB *n* = 1; IIB *n* = 2) were treated with revision surgery, osteosynthesis and amputation respectively. It is important to notice that even though individual cases of Felix type B fractures can be handled with ORIF, usually revision surgery is required. Intraoperative fractures were observed in 18.6% (*n* = 19/102) by Felix et al. [13] which confirms our findings of 22.2% (n = 2/9). Both intraoperative fractures were diagnosed with a delay, supporting the assumption of a high rate of underreported cases.

Postoperative fractures are predominantly observed in females and usually associated with a low-energy trauma [13]. Felix et al. figured out that the fracture type and the related proportion of a loose implant is predictive for the treatment success; while Felix type I fractures had the lowest survival rates, Felix type III fractures revealed the highest. Similarly, we observed a complication rate of 67% in type III fractures (*n* = 6), but no adverse event with our Felix type I fracture.

In this content it must be noted, that Felix et al. developed their algorithm for a heterogenous collective retrospectively. The subgroups were also classified regarding their related treatment which makes it difficult to compare the outcome of the fracture types. Patients who underwent immediate revision in the Felix cohort type Ib required later revision in 22.2% (*n* = 6/27), type IIIa were usually treated conservatively and type IIIb delayed for revision for health reasons [13].

Aside from Felix et al., there are only 3 original articles currently reported on the outcome of periprosthetic tibial TKA fractures, all with a limited number of 7–16 patients (Table 2) [13, 17–20]. Another study biomechanically evaluated the treatment options of periprosthetic tibial plateau fractures during UKA [20]. The authors found a significantly higher fracture stability for angle-locking plates compared to cannulated screws [20] which seems to be transferable to tibial TKA fractures.

**Table 2:** Original articles analysing periprosthetic tibial fractures

Author	Year	Title	n	Central message
Our study	2018	Periprosthetic tibial fractures in total knee arthroplasty –an outcome analysis of a challenging and underreported surgical issue	9	Soft tissue management, correct alignment and minimal invasive procedures are important for the outcome of old and osteoporotic patients associated with a high complication rate
Kim et al	2016	Successful outcome with minimally invasive plate osteosynthesis for periprosthetic tibial fracture after total knee arthroplasty.	16	Minimally invasive plate osteosynthesis with locking plates can achieve satisfactory results regarding union, alignment, range of motion and functional outcome
Seeger et al	2013	Treatment of periprosthetic tibial plateau fractures in unicompartimental knee arthroplasty: plates versus cannulated screws.	12	Biomechanical analysis of matched fresh frozen tibiae demonstrating that angle stable plates show significantly higher fracture loads than fixation with cannulated screws and should be preferred
Tabutin et al	2007	Tibial diaphysis fractures below a total knee prosthesis	6	Successful results with intramedullary nailing in osteoporotic bone stock regarding bone healing and knee function
Thompson et al	2001	Periprosthetic tibial fractures after cementless low contact stress total knee arthroplasty.	7	Correct alignment and possible cement fixation regarding tibial component insertion is important in primary TKA as malalignment and osteopenia are risk factors for periprosthetic fractures

Kim et al. could treat 16 tibial TKA fractures (Felix type II *n* = 6, Felix type III *n* = 10) with a locking plate in MIPO technique and achieved satisfactory results [17]. The authors emphasized the importance of a rigid proximal fixation, as fewer than 8 cortices giving purchase to screws showed higher failure rates [17]. Considering the high rate of postoperative wound healing disorders in this fracture entity, the use of MIPO techniques seems favourable.

The aspect of plating can also be extended to the question of single vs double plating respectively mono vs polyaxial locking plates in periprosthetic fractures as mono axial plates often used in combination with double plating was preferred in some cases so far. Hanschen et al. could demonstrate that single plating with polyaxial locking plates in complex distal femur fractures leads to good functional and clinical results [21]. Regarding the soft tissue management as well as the outcome in the treatment of Felix fractures so far, the transfer of this aspect to the tibial side should be well considered.

Similarly, Tabutin et al. treated 6 tibial diaphysis fractures after TKA (all Felix type IIIB) successfully with less invasive intramedullary nailing [18]. Although not applicable on all cases, this offers a less invasive technique when the lateral radiograph shows enough space for the nail between the prosthesis keel and the anterior tibial tuberosity [18].

Thompson et al. described 7 tibial fractures (Felix type I) after changing from a cemented to a cementless TKA, which were successfully treated conservatively (*n* = 3) or with a long cemented stem (*n* = 4) [19]. Risk factors for the occurrence were a preoperative neutral or valgus knee axis and osteopenia whereas age, gender and diagnosis were not [19]. The authors underline the importance of tibial cement fixation and a correct alignment [19]. The importance of correct tibial alignment is confirmed by Felix et al. and our results. Furthermore, several studies on periprosthetic TKA fractures clearly identified a varus malalignment as a risk factor for periprosthetic fractures [22–24].

We further identified 4 case reports in the literature reporting on periprosthetic tibial TKA fractures. Fonseca et al. present the case of periprosthetic tibial fracture (Mayo Clinic type I) associated with a tibial stem fracture [25]. Their finite-element CAD analysis revealed that the implant breakage occurred due to tibial overloading at the plate/stem transition zone [25]. The patient was successfully revised with a longer stem and the authors emphasize the importance of respecting local bone quality. Beharrie et al. combined a long tibial stem in a periprosthetic tibial fracture with additional impaction bone grafting similar as known from acetabular or femoral reconstruction [26, 27].

Similarly, Kumar et al. reported a periprosthetic tibial fracture after lateral UKA following a trivial fall resulting in a loose component and a large tibial bone defect [28]. Revision required long stems as well as proximal structural tibial allograft and the authors emphasized the importance of meticulous analysis and preoperative planning [28]. Furthermore, it has to be noted that UKA goes along with an incidence of 0.2% up to 5% of tibial fractures related to the tibial saw cuts [12, 20, 29].

Surgical treatment of periprosthetic fractures is associated with high rates of adverse events and further revisions, wherefore alternative options should always be considered. Doorgakant et al. treated a Type IIa fracture conservatively with pulsed electromagnetic stimulation [30]. Bone union was achieved after 7 months with asymptomatic fully weight-bearing after 14 months. Although this appears to be a viable therapy, the long immobilization as well as other factors such as the fracture pattern, bone loss, patient biology and general condition have to be respected [30].

Complication rates for periprosthetic fractures in TKA is high and differs depending on type of fracture, degree of osteoporosis and applied implant. Fractures at the tibia are connected with a clearly higher rate of adverse events than those at the femur including non-union, malalignment, re-fractures, PJI, arthrofibrosis and implant failure [31, 32]. The outcome is further related to the fracture location, with fractures distal to the implant (Felix type III) revealing a 5-year-survival rate of 87%, while the rate decreases to 51% and 2.5% for Felix type I and II. The high rates of implant failure after type I and II fractures underline the difficulty of treating these periprosthetic fractures [13]. Our data can also confirm the results of Felix et al. reporting a high rate of adverse events and further revisions.

Unfortunately, the low incidences and the poor representation in the literature currently makes it impossible to present statistical valid data for this serious medical problem. Burnett et al. assume that the increasing numbers of TKA together with the longer implant and patient survival will clearly increase the number of periprosthetic fractures. This will be further aggravated by the demographic changes with patients presenting a more complex medical background including multiple revisions or PJI amongst other medical side diagnoses which will additionally increase the preexisting high risk for complications. The conversion rate to arthrodesis and amputation shows the huge impact of those fractures and sometimes can also be a treatment option in the first place for old and multimorbid patients, especially considering the high number of wound healing disorders and infection in this and other studies.

The impact of tibial TKA fractures is further reflected by the low functional outcome according to our outcome scores. In this context, careful soft tissue management and if applicable minimal invasive procedures seem advantageous. However, we could also demonstrate that malalignment after osteosynthesis in periprosthetic fractures is a risk factor for further complications and thus should be avoided. Altogether, the impact and complication rate of periprosthetic tibial TKA fractures suggest that this entity should be treated in a centre with expertise in both revision and arthroplasty and traumatology. Further studies are needed to give more evidence regarding the treatment strategy and outcome of the single fracture entities.

## Conclusions

Periprosthetic tibial fractures are less common and only insufficiently reported in the current literature compared to periprosthetic fractures of the distal femur. These fractures are predominantly recorded in old patients with reduced bone stock and show a high complication rate. In our study we can confirm the classification and treatment options according to Felix et al. Nevertheless, individual solutions must be considered facing epidemiological developments and complex settings in revision arthroplasty. In case of plate fixation, correct alignment and soft tissue management are considerable factors for the postoperative outcome and should favour minimal invasive procedures if possible. Further studies are required to properly evaluate and address periprosthetic tibial fractures.

## Abbreviations

MIPO: Minimally invasive plate osteosynthesis
ORIF: Open reduction and internal fixation
PJI: Periprosthetic joint infection
TKA: Total knee arthroplasty
UKA: Unicompartimental knee arthroplasty
